# LncRNA ACART protects cardiomyocytes from apoptosis by activating PPAR‐γ/Bcl‐2 pathway

**DOI:** 10.1111/jcmm.14781

**Published:** 2019-11-20

**Authors:** Hao Wu, Haixia Zhu, Yuting Zhuang, Jifan Zhang, Xin Ding, Linfeng Zhan, Shenjian Luo, Qi Zhang, Fei Sun, Mingyu Zhang, Zhenwei Pan, Yanjie Lu

**Affiliations:** ^1^ Department of Pharmacology (the State‐Province Key Laboratories of Biomedicine‐Pharmaceutics of China Key Laboratory of Cardiovascular Research Ministry of Education) College of Pharmacy Harbin Medical University Harbin China

**Keywords:** apoptosis, Bcl‐2, cardiac ischaemia‐reperfusion injury, lncRNA, PPAR‐γ

## Abstract

Cardiomyocyte apoptosis is an important process occurred during cardiac ischaemia‐reperfusion injury. Long non‐coding RNAs (lncRNA) participate in the regulation of various cardiac diseases including ischaemic reperfusion (I/R) injury. In this study, we explored the potential role of lncRNA ACART (anti‐cardiomyocyte apoptosis‐related transcript) in cardiomyocyte injury and the underlying mechanism for the first time. We found that ACART was significantly down‐regulated in cardiac tissue of mice subjected to I/R injury or cultured cardiomyocytes treated with hydrogen peroxide (H_2_O_2_). Knockdown of ACART led to significant cardiomyocyte injury as indicated by reduced cell viability and increased apoptosis. In contrast, overexpression of ACART enhanced cell viability and reduced apoptosis of cardiomyocytes treated with H_2_O_2_. Meanwhile, ACART increased the expression of the B cell lymphoma 2 (Bcl‐2) and suppressed the expression of Bcl‐2‐associated X (Bax) and cytochrome‐C (Cyt‐C). In addition, PPAR‐γ was up‐regulated by ACART and inhibition of PPAR‐γ abolished the regulatory effects of ACART on cell apoptosis and the expression of Bcl‐2, Bax and Cyt‐C under H_2_O_2_ treatment. However, the activation of PPAR‐γ reversed the effects of ACART inhibition. The results demonstrate that ACART protects cardiomyocyte injury through modulating the expression of Bcl‐2, Bax and Cyt‐C, which is mediated by PPAR‐γ activation. These findings provide a new understanding of the role of lncRNA ACART in regulation of cardiac I/R injury.

## 1 INTRODUCTION

Ischaemic heart disease is the main cause of human death globally, accompanied by myocardial apoptosis, cardiac fibrosis and hypertrophy. Oxidative stress‐induced apoptosis is the crucial factor in cardiac injury with various causes, such as ischaemia/reperfusion (I/R).^1^ Inhibition of apoptosis is pivotal to protect the heart from diverse damages. Blocking apoptosis‐related signalling pathways/molecules is beneficial for the injured cardiomyocytes and the impaired cardiac function.^2^

Long non‐coding RNAs (lncRNAs) are a class of non‐coding RNAs more than 200 nucleotides in length without protein‐coding potentials.^3^ LncRNAs participate in a number of biological processes and pathophysiological events including cancers and cardiovascular disease.^4^ LncRNAs have been demonstrated to regulate cardiac hypertrophy, mitochondrial function, cardiac fibrosis and apoptosis of cardiomyocyte.^5^ For example, the expression of Meg3 (maternally expressed gene 3) was up‐regulated in mouse injured heart after MI and involved in the regulation of apoptosis via binding to RNA‐binding protein FUS (fused in sarcoma).^6^ However, detailed studies about lncRNAs’ role in regulating myocardial apoptosis are still limited.

Among various apoptosis‐related pathways, members of Bcl‐2 family participate in regulating programmed cell death by mediating intracellular pro‐apoptotic and anti‐apoptotic signals.^7^, ^8^ The Bax protein, a member of the Bcl‐2 family, is crucial in the activation of both intrinsic and extrinsic apoptotic pathways in response to diverse stimuli.^9^ In addition, Cyt‐C is known as a significant mediator of apoptosis and is released from the mitochondrial intermembrane space to the cytoplasm in response to apoptotic stimulations.^10^, ^11^ PPAR‐γ (peroxisome proliferator‐activated receptor‐γ), a member of the nuclear hormone receptor superfamily, has been reported to be a protective molecule in tissue repair and ischaemic injury.^12^, ^13^ PPAR‐γ overexpression prevents cardiomyocytes from apoptosis by up‐regulating Bcl‐2 and decreasing the level of reactive oxygen species.^14^

Our previous study showed that lncRNA NONMMUT030245 was significantly up‐regulated in the heart of mice with myocardial infarction,^15^ and it is highly conserved between mouse and human (https://blast.ncbi.nlm.nih.gov/Blast.cgi). In the present study, the role of lncRNA NONMMUT030245 named as anti‐cardiomyocyte apoptosis‐related transcript (ACART) was explored in cardiomyocyte apoptosis. The data showed that ACART alleviated cardiomyocyte apoptosis and PPAR‐γ/Bcl‐2 pathway is involved in this process, which provide new insight into regulation of cardiomyocyte injury.

## 2 MATERIAL AND METHODS

### 2.1 Animals

In this study, healthy male C57BL/6 mice (20‐25 g) and neonatal mice (1‐3 days old) were purchased from the Animal Center of the Second Affiliated Hospital of Harbin Medical University. Mice were kept under standard conditions for animals (temperature, 21 ± 1°C; humidity, 55%‐60%) and received food and water ad libitum. All experimental procedures were in accordance with the Institutional Animal Care and Use and approved by Committee of the Harbin Medical University.

### 2.2 Mouse model of ischaemia/reperfusion

The healthy adult male C57BL/6 mice were randomly divided into sham‐operated and I/R groups. The animals were anesthetized with intraperitoneal injection of avertin (0.2 g/kg). The mouse heart was exposed by a left‐sided thoracotomy. The left anterior descending coronary artery (LAD) was ligated for 45 minutes with a 7/0 silk thread followed by reopening of the artery for 24 hours.^16^, ^17^, ^18^ The mice in sham group underwent a same procedure, but with no LAD ligation. After I/R procedure, the air was removed from the chest and the surgical wounds were sutured. To prevent infection, 0.1 mL penicillin (4 × 10^5^ U/mL) was applied by intraperitoneal injection. Finally, the animals were placed on a heating pad until full recovery of consciousness.

### 2.3 Neonatal mouse ventricular cells isolation and treatment

Neonatal mouse ventricular cardiomyocytes (NMVCs) were isolated from 1‐ to 3‐day‐old mice with 0.25% trypsin at 37°C and cultured in Dulbecco's modified Eagle medium (DMEM, Hyclone Laboratories, Utah, USA) supplemented with 10% foetal bovine serum (Gibco, California, USA), 100 U/mL penicillin and 100 μg/mL streptomycin.^19^ Cells at 80% confluence were transfected with 100 nmol/L siRNA of ACART (Si‐ACART) or a plasmid carrying ACART sequence (500 ng/mL) for 24 hours. Then, cells were treated with hydrogen peroxide (H_2_O_2_, 100 μmol/L) in serum‐free medium for 24 hours or 48 hours or were incubated in an anoxic chamber with 95% N_2_ and 5% CO_2_ in glucose‐free DMEM for 12 hours, followed by 24 hours of exposure to normoxic conditions and DMEM containing glucose.^20^ Rosiglitazone (5.0 μmol/L) a PPAR‐γ agonist and T0070907 (10.0 nmol/L) a PPAR‐γ antagonist^21^ was added to the medium after transfection of ACART or Si‐ACART, respectively.

### 2.4 Cell transfection

ACART siRNA (Si‐ACART) and negative control (NC) were purchased from GenePharma (Shanghai, China), and the sequences are as follows: Sense of Si‐ACART 5’‐CCAGAAUCCCACACGUCAATT‐3’, antisense 5’‐UUGACGUGUGGGAUUCUGGTT‐3’; NC for Si‐ACART, sense 5’‐UUCUCCGAACGUGUCACGUTT‐3’, antisense 5’‐ACGUGACACGUUCGGAGAATT‐3’. The pcDNA 3.1 plasmid carrying the whole ACART sequence (pcDNA‐ACART) and empty pcDNA 3.1 plasmid (pcDNA‐vector) were constructed by Genechem (Shanghai, China). The pcDNA‐vector was used as the negative control. X‐treme GENE siRNA Transfection Reagent (Roche, Basel, Switzerland) or Lipofectamine 2000 reagent (Invitrogen, Carlsbad, USA) was used for the transfection of siRNA or plasmid into cells according to the manufacturer's protocol.

### 2.5 Cell viability assay

Cell viability was determined by MTT (3‐(4, 5‐dimethylthiazol‐2‐yl)‐2, 5‐diphenyltetrazolium bromide) assay. NMVCs were seeded in 96‐well culture plates with 1 × 10^4^ cells/well. After treatment, 20 μL of MTT (Sigma‐Aldrich, St Louis, USA) was added to each well and incubated with the cells for 4 hours at 37°C. Then, the supernatant was discarded and 150 μL of DMSO was added to dissolve the formazan crystals. The microplate reader (BioTek, USA) was used to measure absorbance value at 490 nm.^22^

### 2.6 Lactate dehydrogenase assay

Lactate dehydrogenase (LDH) was measured by LDH detection kits (Nanjing Jiancheng Bioengineering Institute, Nanjing, China) according to the manufacturer's instructions. The absorbance value of each well was measured with a microplate reader (BioTek, Richmond, USA) at 450 nm.

### 2.7 TUNEL assay

NMVCs apoptosis was measured by terminal deoxynucleotidyl transferase‐mediated dUTP‐biotin nick end labelling (TUNEL) assay using in situ cell death detection kit (Roche, Basel, Switzerland) according to the manufacturer's instructions. The nuclei were stained with 4’,6‐diamidino‐2‐phenylindole (DAPI). The samples were observed under fluorescence microscope (Olympus Corporation, Tokyo, Japan).

### 2.8 Quantitative real‐time PCR

Total RNA samples from heart tissues or cultured cells were extracted using TRIzol reagent (Invitrogen, Carlsbad, USA). The RNA was reverse transcribed with reverse transcriptase (Toyobo, Osaka, Japan). The expression levels of ACART and PPAR‐γ mRNA were carried out on ABI 7500 fast Real‐Time PCR system (Applied Biosystems, Carlsbad, USA) by quantitative real‐time PCR. The results of qRT‐PCR were normalized to the expression of GAPDH. The relative quantitative expression was calculated using method 2^−∆∆Ct^. The primers were synthesized by Introvigen and the sequences are as follows: primers for ACART (forward: 5’‐TTCGTGTGCCCAACTCTCTC‐3’; reverse: 5’‐GGAGGGGAAAAATGCACAGG‐3’), primers for PPAR‐γ (forward: 5’‐CCATTGAGTGCCGAGTCTGT‐3’; reverse: 5’‐GAGACATCCCCACAGCAAGG‐3’), primers for GAPDH (forward: 5’‐GATGCCCCCATGTTTGTGAT‐3’; reverse: 5’‐GGCATGGACTGTGGTCATGAG‐3’).

### 2.9 Western blot analysis

Total protein samples were extracted from NMVCs with RIPA lysis buffer (Beyotime Institute of Biotechnology, Shanghai, China). The total protein (100 μg) was electrophoresed on SDS‐PAGE (12% polyacrylamide gels), and subsequently transferred to nitrocellulose membrane (Millipore, Bedford, USA), and then moved to a solution of skim milk powder for 2 hours at room temperature. The membranes were incubated overnight with the primary antibody for PPAR‐γ (Cat.No. 16643‐1‐AP, Proteintech, Wuhan, China), Bcl‐2 (Cat.No. D17C4, Cell Signaling Technology, Boston, USA), Bax (Cat.No. 50599‐2‐Ig, Proteintech, Wuhan, China), Cyt‐C (Cat.No. ab133504, Abcam, Cambridge, USA) and cleaved caspase‐3 (Cat.No. SAB4503294, Sigma‐Aldrich, California, USA) at 4°C. The Western blot bands were quantified using Odyssey imaging system (LI‐COR, Lincoln, USA) and normalized to β‐actin (Cat.No. TA‐09, ZSGB‐BIO, Beijing, China). The mitochondrial and cytosol proteins were separated using the Cell Mitochondria Isolation Kit purchased from Beyotime (Shanghai, China).

### 2.10 Statistical analysis

All experimental data are expressed as mean ± SD. Statistical significance was estimated by ANOVA or Student's *t* test for multiple group or two group comparisons, using GraphPad Prism 7. *P* < .05 was considered to be statistically different.

## 3 RESULTS

### 3.1 ACART was down‐regulated during cardiomyocyte injury

**Figure 1:**
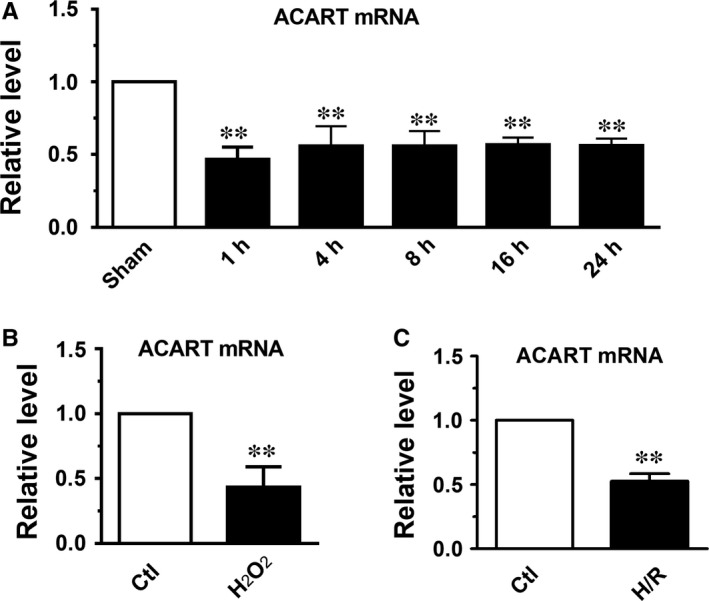
ACART was down‐regulated during cardiomyocyte injury. A, Mice were subjected to myocardial ischaemia for 45 min then the expression level of ACART was assayed by qRT‐PCR at 1, 4, 8, 16 and 24 h after reperfusion. **P < .01 vs Sham, n = 5. B, ACART level was detected in NMVCs treated with 100 μmol/L H_2_O_2_ for 24 h. C, NMVCs were treated with 12 h hypoxia, followed by reoxygenation for 24 h, then ACART level was detected. **P < .01 vs Ctl, n = 4

We have found that ACART was significantly up‐regulated in cardiac fibrotic tissue,^15^ but its function in cardiomyocyte apoptosis was unclear. We firstly examined ACART expression in the hearts of mice subjected to I/R (ischaemia/reperfusion) injury. The qRT‐PCR assay revealed that the level of ACART was significantly decreased in I/R hearts after 1, 4, 8, 16 and 24 hours of reperfusion compared with the sham group (Figure 1A). However, there was no significant difference among these time‐points. In our study, we employed H_2_O_2_ to mimic the pathological change of reactive oxygen species overproduction, which has been widely employed in the field.^23^, ^24^, ^25^ As speculated, the expression of ACART in cultured NMVCs (neonatal mouse ventricular cardiomyocytes) treated with 100 μmol/L hydrogen peroxide (H_2_O_2_), an inducer of apoptosis for 24 hours was also down‐regulated (Figure 1B). Meanwhile, cardiomyocytes were treated with 12 hours hypoxia, followed by reoxygenation for 24 hours. After 24 hours reoxygenation, the expression of lncRNA ACART was decreased by 48% compared with the control group (Figure 1C), which was consistent with the results from H_2_O_2_‐treated group. Therefore, in this study, 100 μmol/L H_2_O_2_ was used to simulate ischaemia/reperfusion injury in cardiomyocytes.

### 3.2 Overexpression of ACART mitigated H_2_O_2_‐induced cardiomyocyte injury

**Figure 2:**
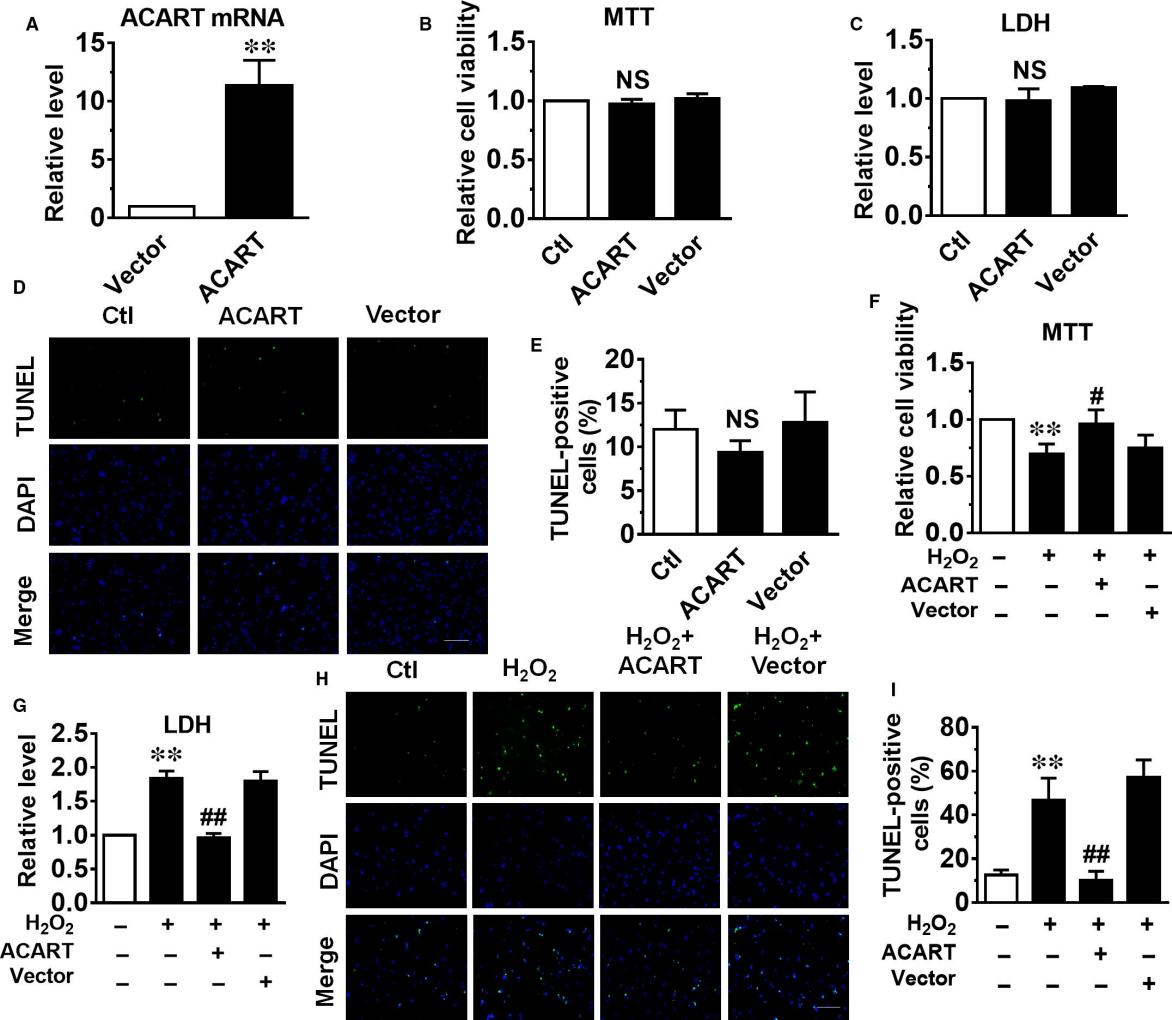
Overexpression of ACART mitigated H_2_O_2_‐induced cardiomyocyte injury. A, The level of ACART was detected by qRT‐PCR in cardiomyocyte transfected with plasmid carrying ACART sequence. **P < .01 vs Vector, n = 4. B, Cell viability of cardiomyocyte was detected by MTT assay. C, LDH release determined by LDH assay. D, Representative images of TUNEL staining. Green fluorescence showed TUNEL‐positive cardiomyocytes; blue showed nuclei of total cells. Scale bar, 100 μm. E, Statistical analysis of TUNEL results. NS, non‐significant. F, ACART inhibited the cell viability decrease induced by H_2_O_2_. G, ACART overexpression restrained H_2_O_2_‐induced LDH release. H and I, ACART mitigated H_2_O_2_‐induced cardiomyocyte apoptosis that was tested by TUNEL assay. **P < .01 vs control, #*P* < .05 vs H_2_O_2_ + Vector, ##*P* < .01 vs H_2_O_2_ + Vector, n = 3‐5

To test the effects of ACART manipulation on cardiomyocyte injury, the effects of ACART overexpression on cardiomyocyte injury were evaluated. We transfected the NMVCs with a plasmid carrying ACART sequence and the expression level of ACART was increased by about 11‐fold (Figure 2A). However, ACART overexpression did not affect cardiomyocyte viability, LDH releasing and cell apoptosis (Figure 2B‐E). We then tested whether ACART overexpression play a role in H_2_O_2_‐induced cardiomyocyte apoptosis. After 24 hours of transfection with the ACART plasmid, we treated NMVCs with 100 μmol/L H_2_O_2_ for 24 hours and detected cell injury. The MTT assay showed that overexpression of ACART inhibited the reduction of cell viability induced by H_2_O_2_, while transfection of empty vector produced no such effect (Figure 2F). Overexpression of ACART dramatically reduced the release of LDH stimulated by H_2_O_2_ (Figure 2G). TUNEL experiments also showed that transfection of ACART plasmid significantly decreased H_2_O_2_‐induced apoptosis in NMVCs (Figure 2H,I).

### 3.3 Knockdown of ACART induced cardiomyocyte apoptosis

**Figure 3:**
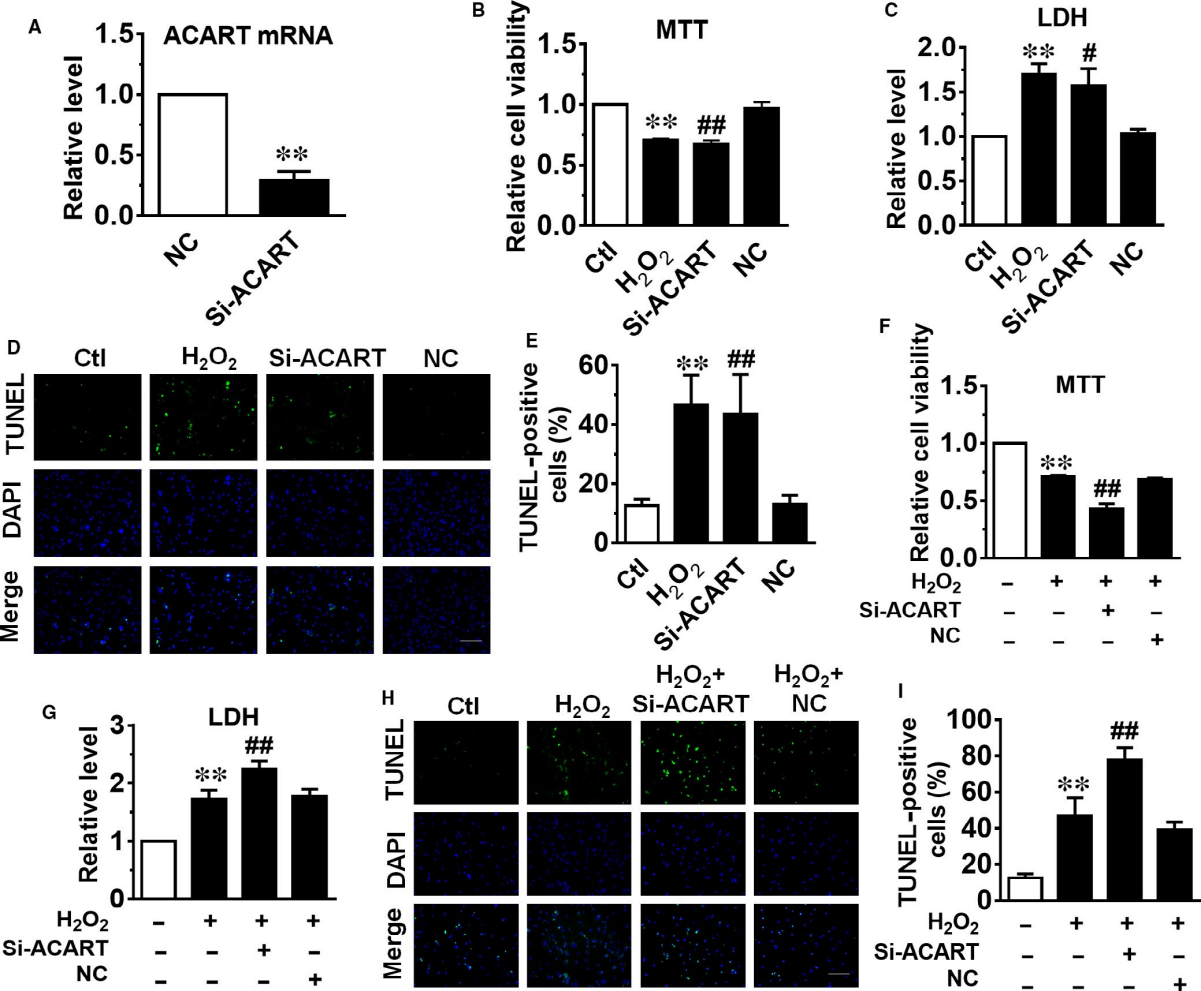
Knockdown of ACART induced cardiomyocyte apoptosis. A, ACART level was detected by qRT‐PCR in NMVCs transfected with Si‐ACART or negative control (NC) for 24 h. **P < .01 vs NC. B, Cell viability was determined by MTT assay after 24 h transfection of Si‐ACART. C, LDH release levels were detected after 24 h transfection of Si‐ACART. D, Images of apoptotic NMVCs by TUNEL staining in different groups. Scale bar, 100 μm. E, Statistical analysis of percentage of TUNEL‐positive cells. **P < .01 vs Ctl, #*P* < .05 vs NC, ##*P* < .01 vs NC, n = 3‐5. F, NMVCs were transfected with Si‐ACART for 24 h, then treated with 100 μmol/L H_2_O_2_ for 24 h. Si‐ACART further reduced cell viability in the presence of 100 μmol/L H_2_O_2_ as determined by MTT assay. G, Knockdown of ACART increased LDH release level. H, Images of cardiomyocyte apoptosis by TUNEL staining in different groups. Scale bar, 100 μm. I, Statistical analysis of TUNEL‐positive cells. **P < .01 vs control, ##*P* < .01 vs H_2_O_2_ + NC, n = 4‐5

To further verify the regulatory role of ACART in cardiomyocyte apoptosis, we employed the siRNA for ACART (Si‐ACART) to knockdown its expression. Transfection of Si‐ACART in cultured NMVCs reduced ACART level by 60% as examined by qRT‐PCR (Figure 3A). Importantly, knockdown of ACART dramatically decreased cardiomyocyte viability as detected by MTT assay (Figure 3B), and increased LDH release compared with negative control (NC) groups (Figure 3C). We performed TUNEL assay to evaluate cell apoptosis and found that knockdown of ACART significantly increased cardiomyocyte apoptosis (Figure 3D,E). These data indicated that down‐regulation of ACART is detrimental to cardiomyocyte.

Next, we examined the effects of ACART knockdown on NMVCs injury induced by H_2_O_2_. As shown in Figure 3F‐I, H_2_O_2_ treatment for 24 hours reduced cell viability, increased LDH release and promoted cell apoptosis. Intriguingly, knockdown of ACART further exacerbated H_2_O_2_‐induced cell injury as manifested by significantly decreased cell viability, increased LDH release and apoptotic rate.

### 3.4 ACART regulated Bcl‐2‐mediated apoptosis of cardiomyocytes

**Figure 4:**
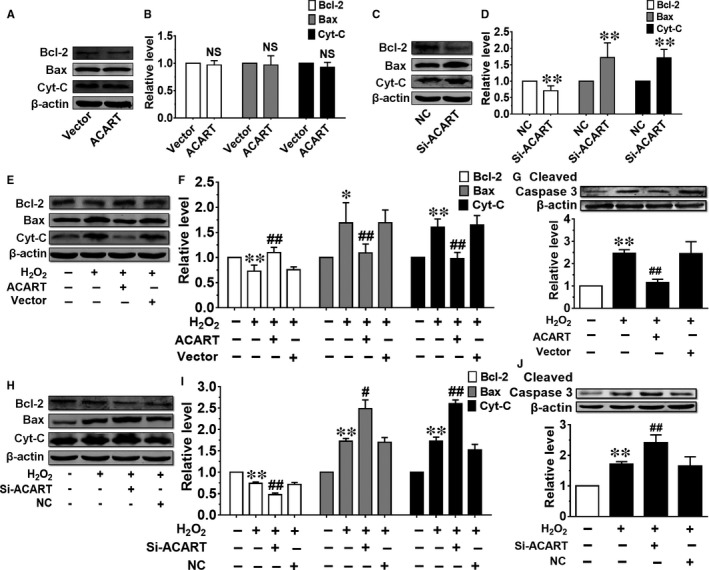
ACART regulated Bcl‐2‐mediated apoptosis of cardiomyocyte. A and B, The protein levels of Bcl‐2, Bax and Cyt‐C were detected by Western blot after ACART plasmid transfection for 48 h. NS, non‐significant. C and D, The Bcl‐2, Bax and Cyt‐C expression levels were detected after transfection of Si‐ACART for 48 h. **P < .01 vs NC, n = 4‐5. E and F, ACART reversed the alterations of Bcl‐2, Bax and Cyt‐C induced by H_2_O_2_ in NMVCs at protein level. G, ACART alleviated the effect of H_2_O_2_ on cleaved caspase‐3 protein expression. H and I, Protein levels of Bcl‐2, Bax and Cyt‐C after silence of ACART detected by Western blot. J, Si‐ACART increased cleaved caspase‐3 level during H_2_O_2_ treatment. *P < .05 vs control, **P < .01 vs control, #*P* < .05 vs H_2_O_2_ + NC, ##*P* < .01 vs H_2_O_2_ + Vector or H_2_O_2_ + NC, n = 4‐6

Bcl‐2, a suppressor of apoptosis, is decreased during apoptosis.^3^ Bax, a pro‐apoptotic protein, is an important factor in the apoptotic signalling pathway.^7^ Cyt‐C is released from the mitochondria into the cytoplasm when apoptosis occurs.^26^ Therefore, the influence of ACART on the expressions of these proteins was explored. We found that ACART overexpression had no effects on protein levels of Bcl‐2, Bax and Cyt‐C (Figure 4A,B). However, Si‐ACART induced significant Bcl‐2 repression, Bax and Cyt‐C enhancement in NMVCs (Figure 4C,D). Importantly, overexpression of ACART counteracted H_2_O_2_‐induced Bcl‐2 reduction and increase of Bax and Cyt‐C expression (Figure 4E,F). We also observed that Cyt‐C was significantly increased in the cytosol after H_2_O_2_ treatment. ACART attenuated this effect, while Si‐ACART enhanced this effect. ACART plasmid and negative control had no effect on the cytosolic Cyt‐C level. Si‐ACART elevated the cytosolic Cyt‐C level in cardiomyocytes without H_2_O_2_ treatment (data not shown). Studies have reported that H_2_O_2_‐induced apoptosis was associated with activation of caspase‐3.^27^, ^28^ As shown in Figure 4G, the expression of cleaved caspase‐3 was significantly increased in H_2_O_2_ group; overexpression ACART for 48 hours significantly down‐regulated the level of cleaved caspase‐3 induced by H_2_O_2_, while the empty plasmid (Vector) had no such effect. In contrast, knockdown of ACART further suppressed Bcl‐2 expression and enhanced Bax, Cyt‐C and cleaved caspase‐3 levels in NMVCs treated with H_2_O_2_ (Figure 4H‐J). These data indicate that ACART regulated expression of apoptosis‐associated factors Bcl‐2, Bax, Cyt‐C and caspase‐3.

### 3.5 PPAR‐γ/Bcl‐2 pathway participated in ACART‐mediated regulation of NMVCs apoptosis

**Figure 5:**
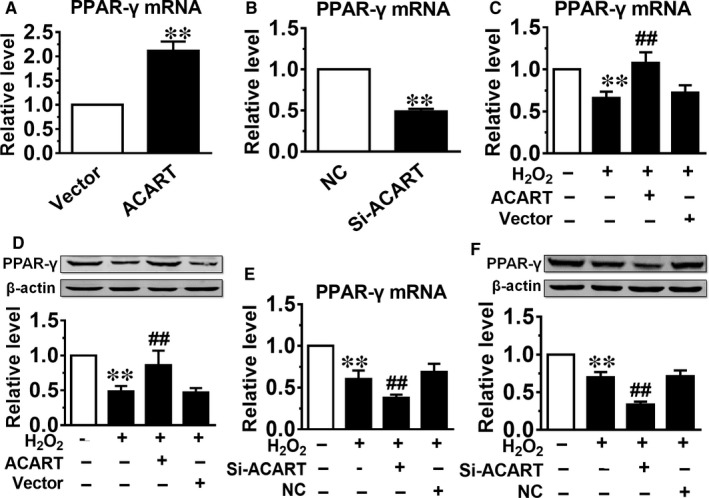
ACART regulated the expression of PPAR‐γ in cardiomyocyte. A, The ACART overexpression increased mRNA level of PPAR‐γ. **P < .01 vs Vector. B, Si‐ACART reduced the mRNA level of PPAR‐γ. **P < .01 vs NC. C, D, E and F, The mRNA and protein expression of PPAR‐γ were detected in H_2_O_2_‐treated NMVCs transfected with the plasmid carrying ACART sequence or Si‐ACART. **P < .01 vs control, ##*P* < .01 vs H_2_O_2_ + Vector or H_2_O_2_ + NC, n = 4‐5

Studies have reported that PPAR‐γ is a critical molecule in protecting cardiomyocytes from apoptosis by regulating the expression of Bcl‐2 family proteins.^29^ We therefore evaluated the potential involvement of PPAR‐γ in ACART‐mediated apoptosis and alteration of Bcl‐2, Bax and Cyt‐C expression. We found that the mRNA level of PPAR‐γ was significantly increased by ACART overexpression (Figure 5A) and reduced by Si‐ACART in NMVCs (Figure 5B). Moreover, overexpression of ACART abrogated the suppression of PPAR‐γ expression in cardiomyocyte treated with H_2_O_2_ at mRNA and protein levels (Figure 5C,D). While knockdown of ACART further repressed the expression of PPAR‐γ in NMVCs with H_2_O_2_ at mRNA and protein levels (Figure 5E,F).

**Figure 6:**
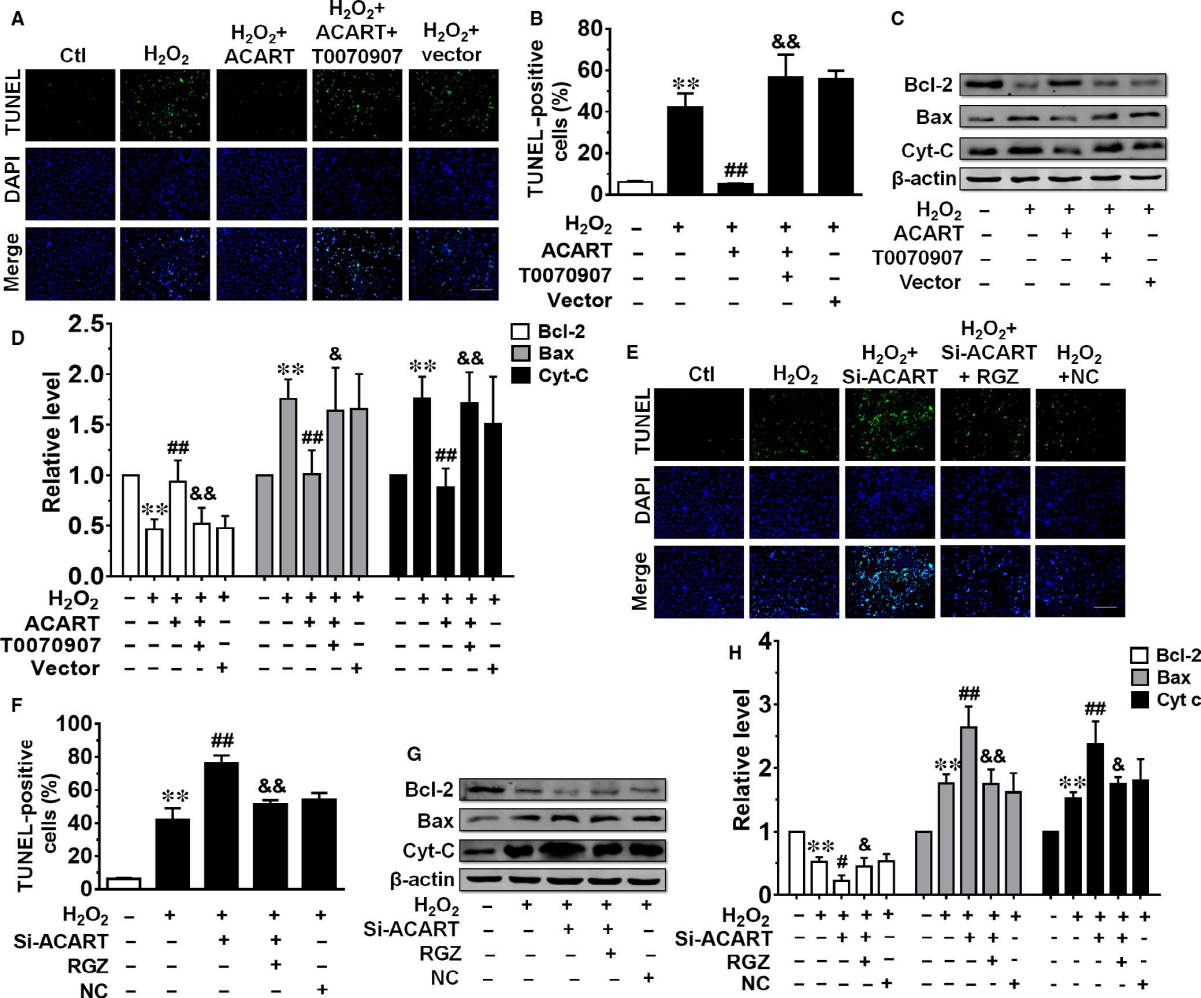
PPAR‐γ/Bcl‐2 pathway participated in ACART‐mediated regulation of NMVCs apoptosis. A and B, T0070907 an antagonist of PPAR‐γ attenuated protective effect of ACART on H_2_O_2_‐induced apoptosis. Scale bar, 100 μm. C and D, T0070907 repressed the effects on Bcl‐2, Bax and Cyt‐C of ACART, protein levels of Bcl‐2, Bax and Cyt‐C were detected by Western blot. E and F, Effects of agonist of PPAR‐γ on TUNEL‐positive cells. Scale bar, 100 μm. G and H, RGZ an agonist of PPAR‐γ restored the alterations of Bcl‐2, Bax and Cyt‐C induced by Si‐ACART in NMVCs. **P < .01 vs control, #*P* < .05 vs H_2_O_2_ + NC, ##*P* < .01 vs H_2_O_2_ + Vector or H_2_O_2_ + NC, &*P* < .05 vs H_2_O_2_ + ACART or H_2_O_2_ + Si‐ACART, &&*P* < .01 vs H_2_O_2_ + ACART or H_2_O_2_ + Si‐ACART, n = 4‐6

To confirm the role of PPAR‐γ in the regulation of ACART on cardiomyocyte apoptosis, both T0070907, an antagonist of PPAR‐γ and Rosiglitazone (RGZ), an agonist of PPAR‐γ were used to treat cardiomyocytes after transfection with plasmid carrying ACART or Si‐ACART. The TUNEL assay showed that T0070907 (10.0 nmol/L) cancelled the protective effects of ACART on NMVC apoptosis upon H_2_O_2_ treatment (Figure 6A,B). The up‐regulation of Bcl‐2 and down‐regulation of Bax and Cyt‐C by ACART in NMVCs treated with H_2_O_2_ were cancelled by co‐administration with PPAR‐γ antagonist T0070907 (Figure 6C,D). Importantly, RGZ (5.0 μmol/L) alleviated NMVCs apoptosis caused by Si‐ACART (Figure 6E,F). In addition, the suppression of Bcl‐2 and enhancement of Bax and Cyt‐C by Si‐ACART were encountered by co‐treatment with PPAR‐γ agonist RGZ (Figure 6G,H). These data indicate that PPAR‐γ/Bcl‐2 pathway participated in the regulation of ACART in NMVCs apoptosis.

Furthermore, we investigated the change of PPAR‐γ, Bcl‐2, Bax and Cyt‐C in mouse hearts subjected to ischaemia/reperfusion injury. Expression of PPAR‐γ and Bcl‐2 was decreased, and the level of Bax and Cyt‐C was increased significantly (data not shown), which are consistent with the in vitro results. Taken together, the results show that ACART regulates cardiomyocyte injury by mediating PPAR‐γ/Bcl‐2 pathway in vitro and in vivo.

## 4 DISCUSSION

In the present study, we characterized lncRNA ACART as a positive regulator of cardiomyocyte apoptosis through PPAR‐γ/Bcl‐2 pathway. We demonstrated that ACART expression is down‐regulated in I/R mouse hearts and H_2_O_2_‐ or hypoxia/reoxygenation‐treated NMVCs; ACART overexpression suppresses NMVC apoptosis under the stimulation of H_2_O_2_ while knockdown of ACART induces cell apoptosis; ACART alleviates cardiomyocyte apoptosis via PPAR‐γ‐mediated expression of Bcl‐2, Bax, Cyt‐C, and caspase‐3. These findings suggest that ACART may act as an important regulator of cardiomyocyte apoptosis.

LncRNAs have received much attention for their roles in pathophysiological processes of cardiovascular diseases.^30^, ^31^ LncRNA CARL (cardiac apoptosis‐related lncRNA) represses mitochondria‐mediated apoptosis through miR‐539/PHB2 pathway in cardiomyocytes.^32^ LncRNA GAS5 (growth arrest‐specific 5) ameliorates MI‐induced cardiomyocyte apoptosis by down‐regulating sema3a (Semaphorin 3a).^33^ LncRNA UCA1 (Urothelial carcinoma‐associated 1) was reported to suppress p27 expression, thereby contributing to cardiomyocyte apoptosis.^34^ LncRNA ACART is 2193 bp in length and located on mouse chromosome 17 (strand: +, chr17: 64, 626, 520‐64, 628, 713). In the present study, we demonstrated that ACART was significantly down‐regulated in ischaemia/reperfusion injured hearts and H_2_O_2_‐ or hypoxia/reoxygenation‐treated cardiomyocyte. Furthermore, our results showed that knockdown of ACART could induce NMVC apoptosis and ACART overexpression attenuated H_2_O_2_‐induced cell injury. This indicates that ACART is potentially involved in regulation of cardiomyocyte apoptosis and pathophysiological processes of ischaemic heart disease.

Cell fate is determined by the balance between pro‐ and anti‐apoptotic factors/molecules, such as Bcl‐2 family members.^35^ Pro‐apoptotic molecule Bax is considered as an inhibitory binding partner of Bcl‐2, it exhibits an extensive amino acid homology with Bcl‐2 and forms heterodimers with Bcl‐2.^36^ The pro‐apoptotic function of Bax is activated in response to deleterious events, resulting in the formation of a channel or other structure in the mitochondrial outer membrane. This is widely accepted that Cyt‐C exits mitochondria to trigger apoptosis through the conduit.^26^, ^37^, ^38^, ^39^ Here, we demonstrated that H_2_O_2_ down‐regulated expression of Bcl‐2 and ACART in NMVCs, while elevates the Bax and Cyt‐C expression. Overexpression of ACART increased Bcl‐2 expression, inhibited Bax and Cyt‐C expression. However, knockdown of ACART produced opposite effects. When apoptosis occurs, Cyt‐C is released from the mitochondria into the cytosol where it participates in caspase activation. ACART attenuated cytosolic Cyt‐C increase after H_2_O_2_ treatment, while Si‐ACART enhanced this effect. These results suggest that Bcl‐2, Bax and Cyt‐C were involved in the regulation of cardiomyocyte apoptosis by ACART.

It has been reported that PPAR‐γ, a ligand‐activated transcription factor protects cardiomyocytes from oxidative stress‐induced apoptosis by up‐regulating Bcl‐2 expression.^14^, ^40^ Importantly, PPAR‐γ activation possesses potent protective actions and has been recently determined as potential therapeutic agents for cardiovascular disease. Our study demonstrates that ACART promoted PPAR‐γ and Bcl‐2 expression and reduced Bax and Cyt‐C expression while T0070907 a PPAR‐γ antagonist abolished the effects of ACART on Bcl‐2, Bax and Cyt‐C expressions. These data imply that ACART possesses the regulatory effects on PPAR‐γ‐mediated expression of Bcl‐2, Bax and Cyt‐C. More importantly, PPAR‐γ antagonist blocked the protective effects of ACART on NMVCs apoptosis; however, PPAR‐γ agonist abrogated cell apoptosis induced by ACART silencing. Taken together, the present study demonstrates that PPAR‐γ/Bcl‐2 pathway is involved in the regulation of ACART in NMVCs apoptosis.

LDH is a soluble cytosolic enzyme and indicator of cell membrane integrity present in most eukaryotic cells.^41^ It cannot penetrate the cell membrane under physiological conditions; however, when cell membrane is damaged, LDH leaks from the intracellular substance to the extracellular matrix or culture supernatant.^42^, ^43^ H_2_O_2_‐induced oxidative stress results in membrane lipoperoxidation and membrane destabilization leading to cell membrane permeability increased.^44^, ^45^ Therefore, when cardiomyocytes are stimulated by H_2_O_2_ to induce cell damage, the release of LDH to the culture supernatant is increased. Apoptosis is characterized by cell shrinkage, DNA fragmentation, chromatin condensation and apoptotic bodies, while the cell membrane is intact. In our study, both LDH release and TUNEL‐positive cells were increased in cardiomyocytes treated with H_2_O_2_, which was alleviated by the overexpression of ACART, indicating that ACART influence both cell membrane damage and apoptosis.

The potential limitations of the present study are as follows: Firstly, the exact mechanism of PPAR‐γ regulation by ACART has not yet been explored. Based on lncRNA biological function, ACART may regulate PPAR‐γ expression at gene transcriptional or/and post‐transcriptional levels. Therefore, it could not be ruled out that other mechanisms are included in regulation of cardiac I/R injury by ACART. Secondly, the effects of ACART on cardiac injury and function in vivo need to be evaluated in future study.

In summary, our findings demonstrate that ACART protects cardiomyocyte from apoptosis via at least PPAR‐γ/Bcl‐2 pathway and provide new understanding of the role of lncRNA in regulation of cardiomyocyte apoptosis.

## CONFLICT OF INTEREST

The authors confirm that there are no conflicts of interest.

## AUTHORS’ CONTRIBUTIONS

Hao Wu, Haixia Zhu, Yuting Zhuang, Jifan Zhang, Xin Ding, Linfeng Zhan, Shenjian Luo, Qi Zhang performed the research; Yanjie Lu, Zhenwei Pan designed the research study; Fei Sun, Mingyu Zhang analysed the data; Haixia Zhu, Yanjie Lu, Zhenwei Pan, Yuting Zhuang wrote the paper.

## Data Availability

The data that support the findings of this study are available from the corresponding author upon reasonable request.
